# The predictive value of creatinine clearance for mortality in patients undergoing revascularization

**DOI:** 10.1186/s13019-021-01502-1

**Published:** 2021-05-01

**Authors:** Eilon Ram, Pazit Beckerman, Amit Segev, Nir Shlomo, Abigail Atlas-Lazar, Leonid Sternik, Ehud Raanani

**Affiliations:** 1 Department of Cardiac Surgery, Leviev Cardiothoracic and Vascular Center, 52621 Tel Hashomer, Israel; 2 Sheba Medical Center, affiliated to the Sackler School of Medicine, Tel Aviv University, Tel Aviv, Israel; 3 Department of Nephrology, Leviev Cardiothoracic and Vascular Center, 52621 Tel Hashomer, Israel; 4 Department of Cardiology, Leviev Cardiothoracic and Vascular Center, 52621 Tel Hashomer, Israel; 5 Maccabi Health Services, Tel Aviv, Israel

**Keywords:** Ischemic heart disease, Renal function, Glomerular filtration rate, Creatinine clearance

## Abstract

**Background:**

Renal function plays a significant role in the prognosis and management of patients with multi-vessel coronary artery disease (CAD) referred for revascularization. Current data lack precise risk stratification using estimated glomerular filtration rate (eGFR) and creatinine clearance.

**Methods:**

This prospective study includes a three-year follow-up of 1112 consecutive patients with multi-vessel CAD enrolled in the 22 hospitals in Israel that perform coronary angiography.

**Results:**

The Mayo formula yielded the highest mean eGFR (90 ± 26 mL/min per 1.73m^2^) and chronic kidney disease-epidemiology collaboration (CKD-EPI) the lowest (76 ± 24 mL/min per 1.73m^2^). Consequently, the Mayo formula classified more patients (56%) as having normal renal function. There was a significant and strong correlation between the values obtained from all five formulas using Cockcroft-Gault as the reference formula: Mayo: *r* = 0.80, *p* < 0.001; CKD-EPI: *r* = 0.87, *p* < 0.001; modification of diet in renal disease (MDRD): *r* = 0.84, *p* < 0.001; inulin clearance-based: *r* = 0.99, *p* < 0.001). Multivariable analysis demonstrated that decreased renal function is an independent predictor of 3-year mortality in all five formulas, with risk increasing by 15–25% for each 10-unit decrease in eGFR. Despite the similarities between the formulas, the ability to predict mortality was highest in the Mayo formula and lowest in MDRD.

**Conclusions:**

Our data suggest that while the Mayo formula is not currently recommended by any nephrology guidelines, it may be an alternative formula to predict mortality among patients with multivessel CAD, including to the widely used MDRD formula.

**Supplementary Information:**

The online version contains supplementary material available at 10.1186/s13019-021-01502-1.

## Background

Renal disease, particularly chronic kidney disease (CKD), is a common comorbidity in patients with ischemic heart disease, and is associated with worse short- and long-term clinical outcomes [1, 2]. Patients with CKD demonstrate accelerated atherosclerosis and increased risk for multi-vessel coronary artery disease (CAD), which is the most common cause of death in CKD patients [3]. Thus, an accurate definition of CKD status and staging is critical for risk stratification and management of CAD patients [4].

Renal function on admission, assessed by estimated glomerular filtration rate (eGFR) or creatinine clearance (CrCl) is an independent predictor of both short- and long-term mortality in patients undergoing coronary revascularization [5].

There are a number of equations used for GFR estimation and CKD staging. For decades, the Cockcroft-Gault (CG) equation was the most commonly used method for estimating kidney function. Recently, however, due to inherent limitations of the CG, several newer equations have been developed [6–8]. The Modification of Diet in Renal Disease (MDRD) and, more recently, the CKD Epidemiology Collaboration (CKD-EPI) equation, are the most extensively used of the newer formulas. The Mayo Clinic quadratic equation [9] and the inulin clearance-based (IB) eGFR equation [10] were developed in an attempt to better estimate GFR in patients with preserved kidney function.

Current literature fails in providing adequate data regarding the ability of these five formulas to predict outcomes in patients with multivessel CAD undergoing either percutaneous coronary intervention (PCI) or coronary artery bypass grafting (CABG). We aim to evaluate the performance of all five eGFR formulas in predicting 3-year all-cause mortality in a ‘real-world’ cohort of patients with multivessel CAD.

## Methods

### Study design and population

Patients were enrolled and prospectively followed-up in the Multivessel CAD Israeli Registry, details of which have been previously reported [11, 12]. In brief, this registry includes 1112 consecutive adult patients from the 22 hospitals in Israel that perform coronary angiography. All patients had multivessel CAD (Fig. S1). The study was approved by the Institutional Review Board of each of the participating centers and all patients provided informed consent.

### Assessing renal function

We assessed five different eGFR formulas, based on the initial creatinine upon admission prior to pre-procedural hydration or contrast. The formulas used were: CG, MDRD, CKD-EPI, Mayo, and IB (Table 1) (http://www.israc.gov.il/?CategoryID=242).

**Table 1:** Estimated glomerular filtration rate equations

**Cockcroft–Gault (CG) equation:**	
[(140 – Age) × Weight (kg) × (0.85 if female)] / 72 × SCr SCr in milligrams per deciliter	
**Modification of Diet in Renal Disease (MDRD) equation:**	
186 × SCr^− 1.154^ × Age^− 0.203^ × (1.201 if black) × (0.742 if female) SCr in milligrams per deciliter	
**Chronic Kidney Disease Epidemiology Collaboration (CKD-EPI) equation:**	
a × (SCr/b)^c^ × (0.993)^age^ Where the variable “a” takes on the following values based on race and sex: • Black: women = 166; men = 163 • White/other: women = 144; men = 141 The variable “b” takes on the following values based on sex: • Women = 0.7 • Men = 0.9 The variable “c” takes on the following values based on sex and creatinine measurement: • Women: ○ if SCr ≤0.7 mg/dL ➔ − 0.329 ○ if SCr > 0.7 mg/dL➔ − 1.209 • Men: ○ if SCr ≤0.9 mg/dL ➔ − 0.411 ○ if SCr > 0.9 mg/dL = − 1.209 SCr in milligrams per deciliter	
**Mayo Clinic quadratic equation:**	
exp [1.911 + 5.249/SCr − 2.114/SCr^2^–0.00686 × Age − (0.205 if female) If SCr < 0.8 mg/dL than SCr = 0.8. SCr in micromolar	
**Inulin clearance–based (IB) eGFR equation:**	
[(155 − Age) × Weight (kg) / Scr] × (0.85 if female) SCr in micromolar	

*SCr* Serum creatinine (in milligrams per deciliter)

Patients were categorized into five levels of renal function based on the calculated eGFR: no renal impairment (> 90 mL/min per 1.73m^2^), mild renal dysfunction (60–90 mL/min per 1.73m^2^), moderate renal dysfunction (30–59 mL/min per 1.73m^2^), severe renal dysfunction (15–29 mL/min per 1.73m^2^) and kidney failure (< 15 mL/min per 1.73m^2^). The US National Kidney Foundation criteria were adapted for significant renal dysfunction definition as an eGFR of < 60 mL/min per 1.73m^2^ [13].

### Statistical Analysis

Data are presented as mean ± standard deviation. Categorical variables are given as frequencies and percentages. A chi-square test was used for comparison of categorical variables between different renal function groups. Student’s t-test was performed for comparison of continuous variables.

The correlation between CG, MDRD, Mayo, IB and CKD-EPI formulas was tested using Pearson’s correlation coefficient. We performed a Bland and Altman analysis to assess the agreement between values derived from each of the other formulas and the values obtained from the CG formula.

Survival analysis was done using the Kaplan-Meier method, and statistical differences between predefined renal dysfunction groups were tested using the log-rank test. Multivariate Cox proportional hazard modeling was used to identify factors associated with mortality risk at follow-up. Candidate factors appear in Table 2.

**Table 2:** Patient characteristics by the renal function categories

	eGFR ≤60 (*N* = 126)	Discordant eGFR (*N* = 203)	eGFR > 60 (*N* = 783)	*p*-value
Age (years) (mean ± SD)	74 ± 10	73 ± 9	62 ± 10	< 0.001
Gender (male) (%)	86 (68)	136 (67)	663 (85)	< 0.001
Hypertension (%)	119 (94)	170 (84)	530 (68)	< 0.001
Previous PCI (%)	45 (36)	75 (37)	267 (34)	0.751
COPD (%)	11 (9)	20 (10)	47 (6)	0.116
Diabetes (%)	82 (65)	98 (48)	321 (41)	< 0.001
Hemodialysis (%)	5 (4)	0 (0)	0 (0)	< 0.001
Hyperlipidemia (%)	102 (82)	152 (76)	562 (73)	0.087
Smoking (%)	19 (15)	40 (20)	236 (30)	< 0.001
CHF (%)	23 (19)	29 (14)	64 (8)	< 0.001
Prior CVA/TIA (%)	16 (13)	36 (18)	51 (6)	< 0.001
Atrial fibrillation (%)	12 (10)	24 (12)	46 (6)	0.008
SYNTAX score (mean ± SD)	23 ± 9	24 ± 11	22 ± 10	0.026
Body mass index (Kg/m^2^) (mean ± SD)	28 ± 4.7	28.3 ± 7.1	28.9 ± 5.1	0.138
Medical treatment				
Aspirin (%)	93 (77)	149 (76)	519 (70)	0.121
Beta blockers (%)	82 (66)	122 (60)	367 (48)	< 0.001
ACE-I (%)	62 (49)	97 (48)	343 (45)	0.488
Statins (%)	97 (78)	150 (74)	514 (67)	0.012
Anti-hyperglycemic (%)	41 (37)	62 (34)	206 (29)	0.184
Laboratory on admission				
Hemoglobin (mean ± SD)	12 ± 1.7	13.1 ± 1.7	13.9 ± 1.6	< 0.001
Urea (mean ± SD)	62.7 ± 49.8	32.6 ± 17.3	27 ± 13.2	< 0.001
Creatinine (mean ± SD)	2.57 ± 2.21	1.17 ± 0.2	0.87 ± 0.19	< 0.001
HbA1C (mean ± SD)	7.4 ± 2.1	6.7 ± 1.6	7.1 ± 2	0.322
Ethnicity				0.192
Israeli Jews (%)	107 (85)	174 (86)	613 (78)	
Israeli Arabs (%)	19 (15)	29 (14)	156 (20)	
Others (%)	0 (0)	0 (0)	13 (2)	

*eGFR* Estimated glomerular filtration rate, *SD* Standard deviation, *PCI* Percutaneous coronary intervention, *COPD* Chronic obstruction pulmonary disease, *CHF* Congestive heart failure, *CVA* Cerebrovascular accident, *TIA* Transient ischemic attack, *ACE-I* Angiotensin converting enzyme inhibitor

To evaluate the ability of the formulas to predict all-cause mortality, we estimated receiver operating characteristic (ROC) curves and area under the curve (AUC) with 95% confidence interval (CI) using corresponding logistic models. Hosmer-Lemeshow goodness-of-fit test was used to assess model suitability. Furthermore, to predict the benefit incurred by the addition of a GFR formula to a baseline model of mortality prediction, we estimated net reclassification improvement (NRI) and integrated discrimination improvement (IDI). Using binary logistic regression, we computed predicted risk for 3-year mortality from a baseline model without GFR (age, diabetes, history of congestive heart failure, atrial fibrillation, previous stroke, SYNTAX score, chronic obstructive pulmonary disease, hypertension, sex) and a similar model that included GFR (for each formula separately). For calculation of the NRI, rescaled individual predicted risk from baseline and GFR models were compared in three pre-specified risk thresholds; low (< 10%), intermediate (10–20%) and high risk (> = 20%). IDI and relative IDI were computed similarly to NRI, albeit without pre-specified risk thresholds.

Statistical significance was assumed when the null hypothesis could be rejected at *P* < 0.05. All *P*-values are the results of two-sided tests. Statistical analyses were conducted using R (version 3.4.1).

## Results

### Baseline study population characteristics

The mean age of the cohort was 66 ± 11 years, with a male majority of 80%. Among the 1112 patients with multivessel CAD included in the registry, 126 (11.3%) had significant renal impairment (GFR ≤60 mL/min per 1.73m^2^) according to all five formulas (MDRD, Mayo, CKD-EPI, CG, and IB formulas), and 783 (70.4%) had non-significant renal impairment (GFR > 60 mL/min per 1.73m^2^) according to all five formulas. Of the remaining 203 patients (18.3%), the eGFR was discordant and shifted between < 60 mL/min per 1.73m^2^ and > 60 mL/min per 1.73m^2^ depending on the formula used. “Discordant eGFR” was used when a patient was considered to have normal renal function by at least one formula and abnormal renal function by at least one formula. Patients with normal renal function tended to be younger with fewer cardiovascular risk factors such as diabetes, hypertension, history of stroke, history of congestive heart failure, atrial fibrillation and lower SYNTAX scores (Table 2). There were 5 patients on hemodialysis prior to intervention, all were in the significant renal impairment group, and all underwent revascularization by CABG.

CABG was used as the revascularization strategy in 50% of the significant renal impairment group, 51% of the discordant group and 54% of the non-significant renal impairment group. Compared with PCI patients, those who underwent CABG were more likely to be male (83% vs. 77%, *p* = 0.013), had a higher frequency of diabetes (49% vs. 42%, *p* = 0.026), and were more likely to have had a prior stroke (12% vs. 7%, *p* = 0.014). Those who underwent CABG also had higher mean SYNTAX scores (27 ± 9 vs. 18 ± 8, *p* < 0.001), reflective of more complex CAD. However, PCI-treated patients were more likely to have had prior PCI (40% vs. 30%, *p* = 0.001), prior myocardial infarction (32% vs. 25%, *p* = 0.007) and a history of congestive heart failure (12% vs. 8%, *p* = 0.046), than those treated with CABG.

### Estimated glomerular filtration rate

With all five formulas, only a minority of patients had severe renal impairment or kidney failure. The prevalence of patients with renal failure (eGFR < 60 ml/min/1.73m^2^) at baseline was 12.9% by the Mayo equation, 20.9% by MDRD, 22.8% by CG, 22.9% by IB and 24.6% by the CKD-EPI equation.

The mean eGFR values on admission were in the mild renal dysfunction range for both CABG and PCI patients with no differences in eGFR between the two groups (Fig. S2). For both CABG and PCI patients, the Mayo formula yielded the highest mean value (89.8 ± 27.1 and 90.6 ± 25.4 mL/min per 1.73m^2^, *p* = 0.623; respectively) and CKD-EPI the lowest (75.4 ± 24.7 and 76.6 ± 23.4 mL/min per 1.73m^2^, *p* = 0.402; respectively). Notably, the Mayo included more patients with normal renal function (*N* = 625, 56%) and thus fewer with mild (*N* = 343, 31%) and moderate renal dysfunction (*N* = 103, 9%) (Fig. S2, Table 3). This finding was consistent in both the CABG and PCI subgroups.

**Table 3:** Distribution of eGFR according to the five different formulas

	CKD-EPI	MDRD	Mayo	IB	CG
Mean eGFR (mean ± SD)	76 ± 24	83 ± 32	90 ± 26	86 ± 35	88 ± 36
eGFR > 90	359 (32%)	397 (36%)	625 (56%)	464 (42%)	490 (44%)
eGFR 60–90	480 (43%)	483 (44%)	343 (31%)	393 (35%)	369 (33%)
eGFR 30–59	224 (20%)	191 (17%)	103 (9%)	215 (19%)	212 (19%)
eGFR 15–29	27 (2%)	16 (1%)	18 (2%)	25 (2%)	26 (2%)
eGFR < 15	19 (2%)	18 (2%)	20 (2%)	14 (1%)	14 (1%)

*eGFR* Estimated glomerular filtration rate, *SD* Standard deviation, *CKD-EPI* Chronic kidney disease epidemiology collaboration, *MDRD* Modification of diet in renal disease, *IB* Inulin clearance-based equation, *CG* Cockcroft-Gault

**Fig. 1:**
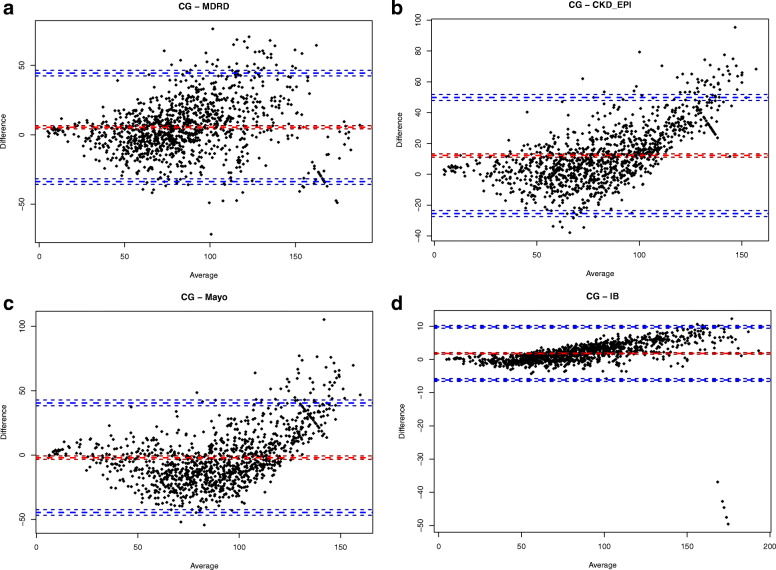
Bland and Altman analysis to assess the agreement between values derived from each formula compared with the CG formula. Red line is for the mean difference and blue lines are for ±1.96 SD. CG = Cockcroft-Gault; MDRD = Modification of Diet in Renal Disease; CKD-EPI=Chronic Kidney Disease Epidemiology Collaboration; IB=Inulin clearance-based equation; SD = Standard deviation

In order to determine whether the CG method, as the reference formula, and the Mayo, CKD-EPI, MDRD, and IB formulas yielded the same results, we calculated the correlation coefficient, and found a strong correlation between the CG formula and each of the other four formulas (Mayo: *r* = 0.80, *p* < 0.001; CKD-EPI: *r* = 0.87, *p* < 0.001; MDRD: *r* = 0.84, *p* < 0.001; IB: *r* = 0.99, *p* < 0.001). Furthermore, in order to assess the agreement between the values obtained from the CG formula and those from each of the other formulas we used the Bland and Altman analyses, which showed good agreement with all formulas used (Fig. 1a-d). The mean ± SD of the eGFR difference between the CG formula (reference) and the Mayo, CKD-EPI, MDRD, and IB formulas were: 2 ± 21.7, 12.2 ± 19.2, 5.4 ± 19.9, and 1.8 ± 4.1 mL/min per 1.73m^2^, respectively.

### Mortality by renal function and post-procedural acute kidney injury

Post-procedural acute kidney injury was more prevalent in the CABG than the PCI group (5.8% vs. 0.9%, *p* < 0.001), and more in the eGFR ≤60 mL/min per 1.73m^2^ than the discordant and eGFR > 60 groups (9.7% vs. 3.9% vs. 1.7%, *p* < 0.001).

**Fig. 2:**
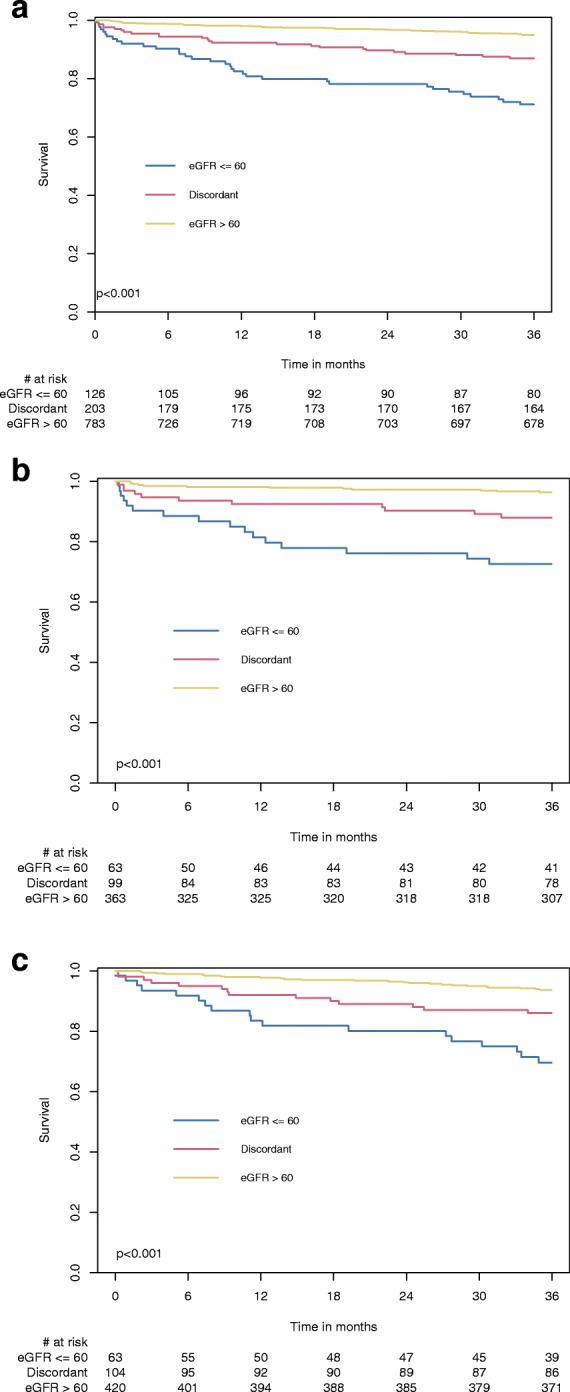
Kaplan-Mayer analysis for survival by the renal function categories. **a** In the entire cohort. **b** Among patients who underwent CABG. **c** Among patients who underwent PCI. eGFR = Estimated glomerular filtration rate; CABG = Coronary artery bypass grafting; PCI = Percutaneous intervention

**Fig. 3:**
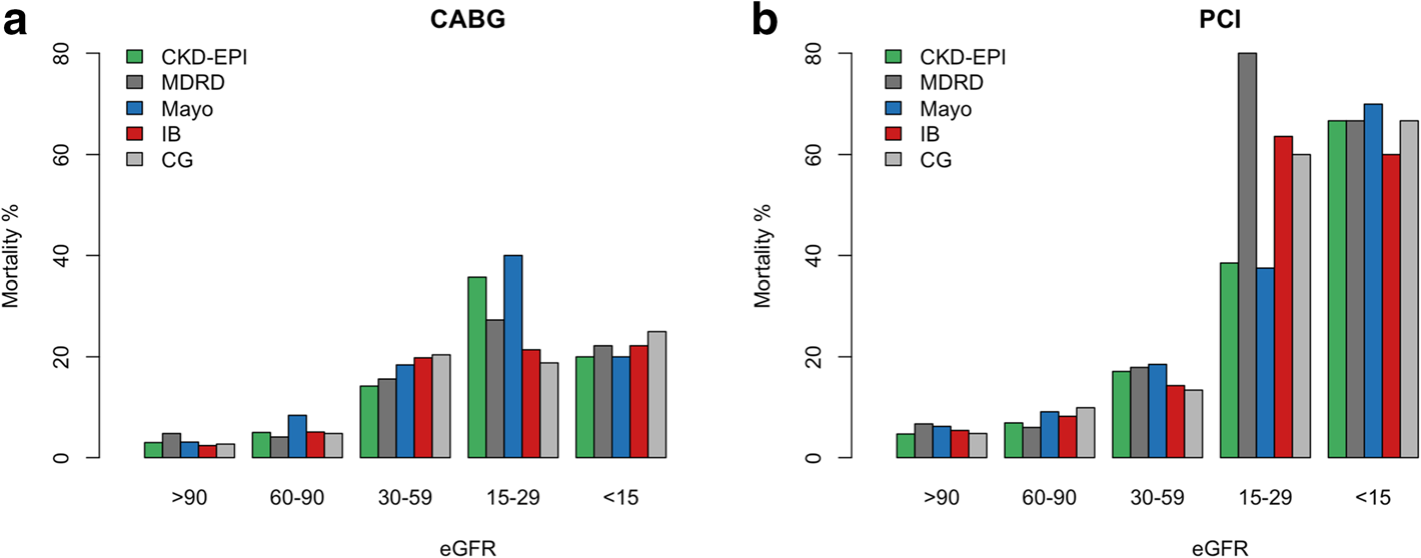
Three-year mortality rates based on renal function status according to the five different formulas. **a** Among patients who underwent CABG. **b** Among patients who underwent PCI. eGFR = Estimated glomerular filtration rate; CKD-EPI=Chronic kidney disease epidemiology collaboration; MDRD = Modification of diet in renal disease; IB=Inulin clearance-based equation; CG = Cockcroft-Gault; CABG = Coronary artery bypass grafting; PCI = Percutaneous intervention

Patients with eGFR ≤60 mL/min per 1.73m^2^ by all five methods had significantly higher 30-day, 1-year and 3-year mortality compared to patients with discordant eGFR, and compared to patients with eGFR > 60 mL/min per 1.73m^2^ by all five methods (5.8% vs. 2.6% vs. 0%, *p* < 0.001; 18.8% vs. 8.2% vs. 2.1%, *p* < 0.001; and 27% vs. 12.3% vs. 4.7%, *p* < 0.001, respectively) (Fig. 2a and Fig. S3). Sub-analysis among CABG and PCI separately revealed similar result (Fig. 2b-c). There were no significant differences in 3-year mortality rates between patients who underwent either CABG or PCI in all three eGFR categories (consistently low eGFR 25% vs. 29%, log-rank *p* = 0.879; discordant eGFR 11% vs. 14%, log-rank *p* = 0.714; consistently high eGFR 3% vs. 6%, log-rank *p* = 0.121; respectively). The overall 3-year mortality rates were not significantly different between the CABG and PCI groups (7.4% vs. 9.7%, log-rank *p* = 0.294). However, the severity of renal impairment correlated with increased mortality (Fig. 3). Multivariable analysis demonstrated that worse renal function is an independent predictor of 3-year mortality for all five formulas (Table 4). Mortality risk was increased by 16–28% for each 10-unit decrease in eGFR, using all five formulas. Other independent predictors of 3-year mortality were older age, chronic obstructive pulmonary disease, previous stroke, history of atrial fibrillation and diabetes.

**Table 4:** Predictors for 3-year mortality^a^. A univariable and multivariable analysis

Formula	Univariable analysis			Multivariable analysis^b^		
	HR	95% CI	***p***-value	HR	95% CI	***p***-value
CKD_EPI	1.35	1.25–1.45	< 0.001	1.28	1.16–1.41	< 0.001
MDRD	1.24	1.15–1.32	< 0.001	1.16	1.07–1.27	0.001
Mayo	1.30	1.22–1.37	< 0.001	1.24	1.14–1.35	< 0.001
IB	1.27	1.18–1.35	< 0.001	1.18	1.08–1.29	< 0.001
CG	1.35	1.25–1.45	< 0.001	1.28	1.16–1.41	< 0.001

*HR* Hazard ratio, *CI* Confidence interval, *CKD-EPI* Chronic kidney disease epidemiology collaboration, *MDRD* Modification of diet in renal disease, *IB* Inulin clearance-based equation, *CG* Cockcroft-Gault, *eGFR* Estimated glomerular filtration rate, *CVA* Cerebrovascular accident, *TIA* Transient ischemic attack, *COPD* Chronic obstruction pulmonary disease

^a^Hazard ratios with 95% CI’s for 3-year mortality (for 10-unit decrements in eGFR)

^b^The covariates included in the model were: age, gender, diabetes, congestive heart failure, history of CVA/TIA, SYNTAX score, atrial fibrillation, hypertension, COPD and post-procedure acute kidney injury

The ability of the five formulas to predict 3-year mortality risk was highest with the Mayo (AUC 0.78 [0.73–0.83]) and lowest with the MDRD formula (AUC 0.75 [0.70–0.80]) (*p* = 0.004). The *p*-value of Hosmer-Lemeshow test was > 0.05 for all models, indicating that they were suitable. The NRI results showed that addition of a GFR formula to the baseline model correctly reclassified approximately one-sixth of patients to a higher predicted risk group (Table 5). IDI analysis demonstrated that, when added to the basic logistic regression model, each of the five formulas improved mortality risk prediction. Among the five formulas, the Mayo had the highest additive effect on mortality prediction (Mayo: rIDI = 26.4%, *p* = 0.001; CG: rIDI = 16.6%, *p* = 0.009; IB: rIDI = 15.2%, *p* = 0.012; CKD-EPI: rIDI = 22.8%, *p* = 0.003; and MDRD: rIDI = 11.7%, *p* = 0.035) (Table 5).

**Table 5:** Discrimination analysis for 3-year mortality according to different GFR formulas

Formula	ROC	Hosmer-Lemeshow test	AIC	NRI	IDI	rIDI
MDRD	0.75 (0.70–0.80)	χ^2^ = 6.8, *p* = 0.553	592.2	14.1% (4.1–24.1%), *p* = 0.006	0.011 (0–0.021), *p* = 0.035	11.7%
CKD-EPI	0.76 (0.71–0.82)	χ^2^ = 9.6, *p* = 0.295	581.9	19.2% (7.9–30.5%), *p* = 0.001	0.025 (0.008–0.041), *p* = 0.003	22.8%
Mayo	0.78 (0.73–0.83)	χ^2^ = 18.2, *p* = 0.644	578.3	15% (3.6–26.4%), *p* = 0.011	0.03 (0.011–0.048), *p* = 0.001	26.4%
IB	0.76 (0.71–0.81)	χ^2^ = 10.6, *p* = 0.225	589.4	16.1% (6–26.1%), *p* = 0.002	0.015 (0.003–0.027), *p* = 0.012	15.2%
CG	0.76 (0.70–0.81)	χ^2^ = 11.2, *p* = 0.190	587.8	17.3% (6.9–27.6%), *p* = 0.001	0.016 (0.004–0.029), *p* = 0.009	16.6%

*GFR* Glomerular filtration rate, *ROC* Receiver operating characteristic, *AIC* Akaike information criterion, *NRI* Net reclassification improvement, *IDI* Integrated discrimination improvement, *rIDI* Relative integrated discrimination improvement, *MDRD* Modification of diet in renal disease equation, *CKD-EPI* Chronic kidney disease epidemiology collaboration equation, *IB* Inulin clearance-based equation, *CG* Cockcroft-Gault equation

## Discussion

This prospective multicenter national registry study describes several cardinal findings regarding evaluation of renal function in patients with multivessel CAD who are referred for revascularization in a ‘real-world’ setting. We have shown that: (1) patients with multivessel CAD and significant renal dysfunction demonstrate increased 3-year mortality; (2) despite the significant and strong correlation between eGFR values using all five formulas, the proportion of patients categorized into the different renal function groups varied considerably, suggesting a significant clinical impact of the GFR formula used; (3) mortality increased as renal function worsened in all renal function categories; (4) patients whose renal dysfunction status shifted from significant to non-significant or vice versa using the different formulas, had mortality rates that were intermediate between those with and without significant renal dysfunction. This suggests that the discordance between the formulas may result from differences in clinically relevant parameters, such as age and weight; and (5) although the MDRD formula is used extensively in clinical practice, it is less accurate in predicting mortality relative to other formulas in patients with multivessel CAD.

The 2011 European Society of Cardiology (ESC) guidelines recommended the MDRD and GC formulas for assessment of renal function in CAD patients [14]. However, according to more recent 2015 ESC guidelines, there is no recommended formula [15]. The CKD-EPI formula is considered the gold standard for eGFR by the 2012 Kidney Disease Improving Global Outcomes guidelines (KDIGO) [16]. In this study, the rate of renal impairment varied from 13 to 25% depending on the formula used, with the choice of formula influencing patient stratification into different renal function categories. We showed that despite this variability in eGFR using different formulas, renal dysfunction remained an independent predictor for mortality with all five formulas. Nevertheless, the absolute mortality rates varied for each renal function category using the different formulas.

Numerous studies have compared the five eGFR formulas outlining important observations. Lin et al. found that the MDRD is more accurate for eGFR calculation in healthy or mildly impaired renal function adults compared with the CG formula [17]. Matsushita et al. showed that the CKD-EPI equation classified fewer individuals as having renal dysfunction and more accurately categorized the risk for mortality than did the MDRD equation [18]. In contrast, however, Carter suggested that the CKD-EPI formula may actually over-estimate renal dysfunction prevalence in elderly patients and showed scarce differences between the CKD-EPI and MDRD formulas [19]. Willems et al. showed that the CG and MDRD formulas effectively predicted mortality, whereas the CKD-EPI formula did not [20]. A recent study by Jo et al. showed that the Mayo equation was the most accurate in predicting post-operative acute kidney injury when comparing the five eGFR formulas in 4125 adult patients undergoing elective cardiovascular surgery [21]. Fu et al. showed that among 1050 patients aged > 60 years with CAD, the CKD-EPI and Mayo equations were significantly superior to the MDRD in predicting 1-year mortality, and the Mayo demonstrated mild superiority over the CKD-EPI equation [22].

Our analysis showed that the Mayo formula offered the most accurate mortality prediction in CAD patients undergoing revascularization, thus confirming the results of previous studies [22]. This finding can be explained by the coefficients and variables included in the equations. As both older age and lower body weight are well-known predictors of outcome in patients with CAD, it follows that a formula accounting for these factors would more accurately predict mortality. While the Mayo formula was found to accurately predict mortality in our cohort, it is less widely used, has not been validated in diverse populations and is not currently recommended by any nephrology practice guidelines. Furthermore, previous reports have shown that the Mayo equation proved inaccurate in type-2 diabetic patients with hyperfiltration [23] or normal renal function [24] and in the very elderly [25, 26].

Interestingly, we have shown that in a ‘real-world’ setting the choice of GFR, as reflected by the different categories (consistently low, discordant and consistently high eGFR), did not affect decision-making regarding the revascularization approach. Indeed, we did not detect any significant difference in the 3-year all-cause mortality by the different revascularization strategies.

### Limitations

This study was based on a national prospective observational registry of patients admitted for multivessel CAD. As such, bias in treatment decision cannot be excluded. Another limitation is the lack of available data on renal dysfunction duration. While our results were adjusted for possible confounding variables, residual confounding cannot be excluded, and the lack of adjustment for variables not captured in the registry may represent a limitation. Data regarding other outcomes such as recurrent myocardial infarction, recurrent revascularization and renal function during the follow-up period were unavailable. Analysis of cardiac events could reinforce the conclusion that significant renal dysfunction upon admission is associated with late cardiac events.

## Conclusions

In patients with multivessel CAD who undergo revascularization with either CABG or PCI, significant renal dysfunction upon admission is associated with mortality, regardless of the eGFR formula used. While the Mayo formula is not currently recommended by any nephrology guidelines, our data suggest that it may be an alternative formula to predict mortality among patients with multivessel CAD. These findings have important implications for everyday clinical practice in risk stratification and management of CAD patients.

## Supplementary Information

**Additional file 1: Figure S1.** Flow chart summary from consent and eligibility, through a 3-year follow-up. CAD = Coronary artery disease; PCI = Percutaneous coronary intervention; CABG = Coronary artery bypass grafting

**Additional file 2: Figure S2.** Distribution of CKD stages determined by eGFR according to the five different formulas. A - Among patients who underwent CABG. B - Among patients who underwent PCI. CKD = Chronic kidney disease; eGFR = estimated glomerular filtration rate; CABG = coronary artery bypass grafting; PCI = percutaneous coronary intervention; CKD-EPI = Chronic Kidney disease Epidemiology Collaboration; MDRD = Modification of Diet in Renal Disease; IB = Inulin clearance–Based equation; CG = Cockcroft–Gault

**Additional file 3: Figure S3.** Kaplan-Mayer analysis for survival by the renal function categories by the different eGFR formulas. eGFR = Estimated glomerular filtration rate; MDRD = Modification of Diet in Renal Disease; CKD-EPI = Chronic Kidney disease Epidemiology Collaboration; IB = Inulin clearance–Based equation; CG = Cockcroft–Gault

## Data Availability

The datasets used and/or analyzed during the current study are available from the corresponding author on reasonable request.

## Abbreviations

AUC: Area under the curve
CABG: Coronary artery bypass grafting
CAD: Coronary artery disease
CG: Cockcroft–Gault
CI: Confidence interval
CKD: Chronic kidney disease
CKD-EPI: Chronic kidney disease Epidemiology Collaboration
CrCl: Creatinine clearance
eGFR: Estimated glomerular filtration rate
IB: Inulin clearance–based
MDRD: Modification of Diet in Renal Disease
NRI: Net reclassification improvement
PCI: Percutaneous coronary intervention
rIDI: Relative integrated discrimination improvement
ROC: Receiver operating characteristic
